# Functional prediction of environmental variables using metabolic networks

**DOI:** 10.1038/s41598-021-91486-8

**Published:** 2021-06-09

**Authors:** Adèle Weber Zendrera, Nataliya Sokolovska, Hédi A. Soula

**Affiliations:** 1 Sorbonne University, 75005 Paris, France; 2 INSERM UMRS1269 NutriOmics, 75013 Paris, France

**Keywords:** Computational biology and bioinformatics, Microbiology, Systems biology

## Abstract

In this manuscript, we propose a novel approach to assess relationships between environment and metabolic networks. We used a comprehensive dataset of more than 5000 prokaryotic species from which we derived the metabolic networks. We compute the scope from the reconstructed graphs, which is the set of all metabolites and reactions that can potentially be synthesized when provided with external metabolites. We show using machine learning techniques that the scope is an excellent predictor of taxonomic and environmental variables, namely growth temperature, oxygen tolerance, and habitat. In the literature, metabolites and pathways are rarely used to discriminate species. We make use of the scope underlying structure—metabolites and pathways—to construct the predictive models, giving additional information on the important metabolic pathways needed to discriminate the species, which is often absent in other metabolic network properties. For example, in the particular case of growth temperature, glutathione biosynthesis pathways are specific to species growing in cold environments, whereas tungsten metabolism is specific to species in warm environments, as was hinted in current literature. From a machine learning perspective, the scope is able to reduce the dimension of our data, and can thus be considered as an interpretable graph embedding.

## Introduction

The rise of sequencing and high-throughput technologies has opened a new era of information and characterisation of previously unknown species. Different techniques have emerged alongside it, to make sense of this new knowledge. Through the use of large metabolic databases such as the Kyoto Encyclopedia of Genes and Genomes (KEGG)^1^, it is possible to have access to genomic, enzymatic, and metabolic information, and deduce some complex interactions happening inside the organisms.

In this context, metabolic networks have been used to study the set of all chemical reactions of organisms in a holistic manner, providing insights into its underlying structure and the different adaptations of these organisms to their environment.

Networks or graphs are mathematical structures linking objects—*nodes*—with *edges*. In the case of metabolic networks, multiple representations can be adopted. Among the most accurate, there is one with metabolites and enzymes as nodes, in which each substrate of a reaction will be linked to the catalysing enzyme, which is in turn linked to the products of the reaction. This is the representation we used when reconstructing our metabolic networks.

In recent years, two main avenues of research have been undertaken to study metabolic networks. The first one studies metabolism structure and properties through network topological measures, such as degree distribution^2–4^, average path length^2–4^, network motifs^3^, and clustering coefficient^3^. The second one relies on the analysis of the fluxes of biomass in metabolism and their dynamics. Various constraint-based methods that try to predict steady-state flux distributions exist, such as Flux Balance Analysis (FBA)^5^. The constraint-based approaches need complete and curated knowledge of fluxes, together with reconstructed state-of-the-art metabolic networks, as it is done, e.g., in ModelSEED^6^. In this paper, we instead follow the first research direction, and are only interested in topological metrics for graph comparison.

Several studies reveal that different metabolic network topological measures—metabolism structural properties—are related to different environmental variables, such as optimal growth temperature^7,8^, oxygen levels^9^, and the type of habitat^10^. However, details on the specific metabolic pathways and mechanisms governing the evolution and adaptation to environment of the organisms are not always considered.

**Figure 1:**
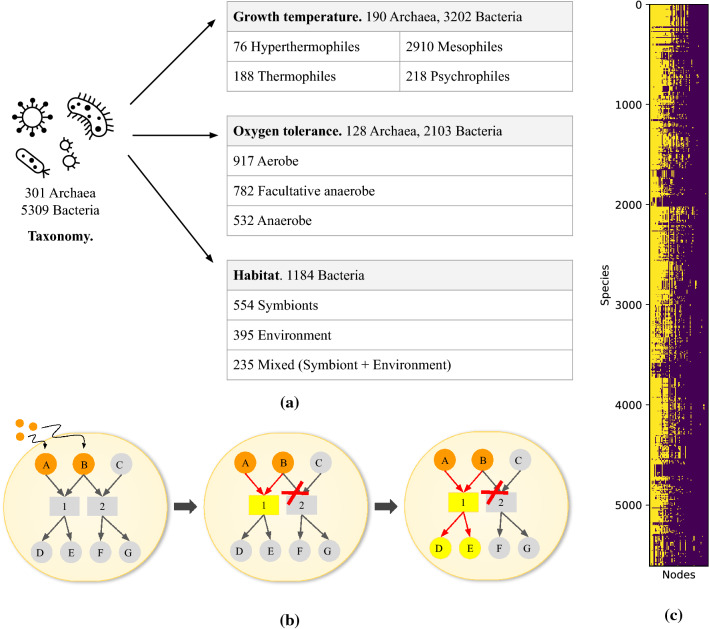
(**a**) Data description. (**b**) Concept of scope of a metabolic network. Letters are metabolites and numbers are reactions. Starting from seed metabolites (orange), the network expands if the substrates of the reactions are present in the expanding network, thereby producing its products. This is the case for reaction 1, which uses A and B to produce D and E, but not for reaction 2, as C is missing. The scope of the metabolic network is the set of all metabolites and reactions in the resulting network (yellow and orange). (**c**) Matrix of nodes in scope per species. In yellow, nodes that are in the scope, in purple the nodes that are out of the scope. Nodes that are in or out of the scope for all species have been removed. Species are sorted in taxonomic order, and 578 nodes are sorted by number of species with node in the scope

In 2005, Handorf, Ebenhöh, and Heinrich presented the concept of scope of a metabolic network^11^. It is based on the concept of network expansion. Starting from seed metabolites from the environment, the network expands if the substrates of the reactions are present in the expanding network, thereby producing its products. This goes on until the network can no longer expand. The scope of the metabolic network is the set of all metabolites and reactions in the resulting network (see Fig. 1b). This network topological measure has the advantage of taking into account the fact that enzymes need all of their substrates to catalyse a reaction and not only one, which is a property specific to metabolic networks that generic graph theory measures do not consider, while also compressing the dimension of our data, and relying purely on network topology. Additionally, it can handle pathway redundancy, depending on the input seed metabolites given to the network.

Generally, graph embeddings in computational biology are based on different strategies (random walk based, deep learning based and factorization based)^12^, and are rarely biologically interpretable^12,13^. In contrast, the scope can be considered as an interpretable graph embedding, since the nodes in the scope can still be understood from a metabolic viewpoint, and are mechanistically related.

In this study we analyse the scopes of a very large dataset of 5610 prokaryotic metabolic networks that we have derived from KEGG. Crossing with other databases, we assess the relationship between the scope and several environment features. In order to detect this relationship, we use machine learning approaches to predict different evolutionary and environmental conditions (taxonomy, growth temperature, habitat, and oxygen tolerance) from their scope. Importantly, as the scope can be considered as an interpretable embedding of our metabolic networks, it will allow us to gain insights into the metabolic processes associated with the different environmental variables.

## Results

### Scope of metabolic networks

The metabolism of living organisms can be described with metabolic networks. In this study, the directed metabolic networks of 301 archaea and 5309 bacteria were derived from KEGG database, with nodes as metabolites and reactions, and edges linking substrates to reaction and reaction to products (see “Methods”).

In the field of systems biology, it is necessary to focus on the complex interactions within an organism and with its environment to be able to understand the complete system. Previous works have shown the strong associations between evolutionary and environmental variables and the underlying topology of the metabolic networks of different organisms^7–9^. However, these approaches do not provide any information on the affected metabolic functions.

To counter this issue, we have chosen to use the scope of a metabolic network, introduced in 2005 by Handorf, Ebenhöh, and Heinrich^11^. We have described the concept on Fig. 1b. This network topological property is specific to metabolic networks, and it is defined as the metabolites and reactions (yellow and orange on the figure) which can recursively be reached from a set of seed compounds (orange). Indeed, starting from the seed compounds, only enzymes with substrates among them will be considered, producing some metabolites which will also be in the scope, and this will go on until no further enzyme and metabolite can be reached (see “Methods”). This approach is purely topological, identifying metabolites that can *potentially* be produced from a set of seed metabolites, and does not rely on biomass fluxes. We would like to emphasize that our main interest here is the interaction between potential metabolic functions described in the scope with other taxonomical and environmental parameters (temperature, habitat and oxygen). We are not interested in the many other properties obtained from classical metabolic networks, e.g., their flux properties and/or biomass optimization. Such information would limit the number of prokaryotic species we are able to use.

The union of the metabolites of three chemically-defined growth mediums used to grow a mesophilic species^14^, a psychrophilic species^15^ and a hyperthermophilic species^16^ are fixed as the seed compounds. A thermophilic medium^17^ was already included, since the union is used as seed for all species. The single medium—the union of mediums—as seed compounds for the scope allows us to extract discriminating properties between the metabolic networks while mitigating potential species-based bias. We built a matrix of nodes in scope per species, removing the nodes that are in the scope for all species or out of the scope for all species, that is shown on Fig. 1c.

For a union of all nodes of all species (6554 nodes), we obtained 578 nodes in the scope, showing that the seed compounds chosen are in fact rather restrictive, and that the dimension of our data is significantly reduced. However, Fig. 1c also reveals the big variability of the scope for each species, with different patterns and also different sizes of the nodes in the scope. As the species in the matrix are sorted taxonomically, blocks of species having similar scopes are usually very close in the tree of life or are even different strains of the same species.

**Figure 2:**
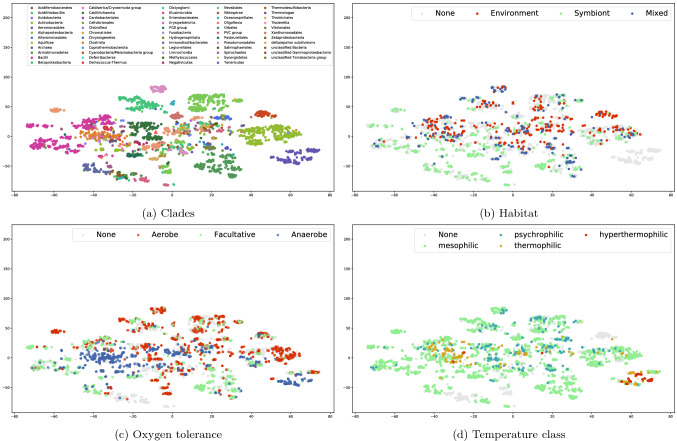
2D t-SNE visualisation. Projection of the nodes in scope per species. The Jaccard distance and a perplexity of 40 were used. Each species has been coloured according to its clade (**a**), habitat (**b**), oxygen tolerance (**c**), and temperature class (**d**)

In order to visualise and to explore the data, we used a t-distributed Stochastic Neighbor Embedding (t-SNE), and plotted our scope per species matrix in two dimensions. The species are characterized by: clades (Fig. 2a), habitat (Fig. 2b), oxygen tolerance (Fig. 2c), and temperature class (Fig. 2d).

The t-SNE visualisation constructs a two-dimensional map that reveals structure at different scales. The method was reported to identify well local dependencies of high-dimensional data, as well as to capture global data structure such as relationships between several clusters. It clearly illustrates the clusters corresponding to all our taxonomic and environmental variables, particularly the clades (Fig. 2a), habitat (Fig. 2b), and oxygen tolerance (Fig. 2c). The results of the growth temperature class (Fig. 2d) are not so clear for lower temperatures (psychrophilic and mesophilic species).

Our results demonstrate the practical utility and very reasonable predictive performance of the scope, since even though the method reduces the data dimensionality drastically, the embedding still carries sufficient information to visualise (in our case, the environmental adaptation), and to explain it.

To investigate whether the scope is able to predict the environmental variables and provide us with additional insights into the functional metabolism, we applied other machine learning approaches, described below.

### Growth temperature analysis

We have growth temperature data for 3392 species out of 5610, illustrated on Fig. 1a.

#### Growth temperature class prediction

First, we train a random forest model to predict the growth temperature classes. Growth temperature can be divided into four temperature classes, from hot to cold, we obtained: 76 hyperthermophiles (HT), 188 thermophiles (T), 2910 mesophiles (M), and 218 psychrophiles (P) (see further details in “Methods”). In order to balance the classes, we both reduced the number of mesophiles to 300 to be on par with the other classes and weighted the objective functions according to sizes in groups.

Then, we executed a 300-fold cross-validation on 1000-tree random forests. For each fold, we randomly chose the 300 mesophiles (for more details, see “Methods”).

**Figure 3:**
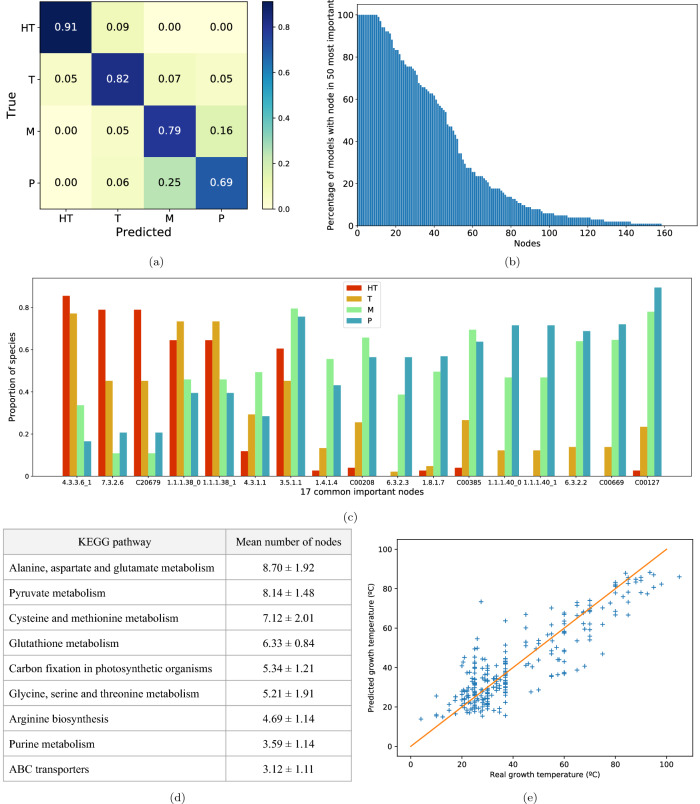
Growth temperature prediction. (**a**) Confusion matrix of a growth temperature class prediction using random forest, mean over 300-fold cross-validation. (**b**) Percentage of models including nodes identified as 50 most predictive nodes, among 300 models (300-fold cross-validation) to predict the growth temperature using the random forest classifier. The 50 most predictive nodes were found applying the Gini impurity to each model. (**c**) Proportion of species per temperature class having 17 most predictive common nodes in their scope. These nodes are ones found in more than 90% of cross validated models (**b**). X.X.X.X codes (X a number) are Enzyme Commission (EC) numbers that are associated with enzymes. We appended “_0”, “_1” to EC numbers to designate different reactions catalysed by the same enzyme. CXXXXX are KEGG database C numbers, associated with metabolites. (**d**) KEGG pathways of the most predictive nodes, and the associated average number of pathway nodes per cross-validation fold, among 50 most predictive nodes. We only show pathways with more than 3 nodes. (**e**) Neural network growth temperature prediction. $R^2=77.09\%$. In orange, $y = x$. *HT* hyperthermophiles, *T* thermophiles, *M* mesophiles, *P* psychrophiles

The average maximal tree depth was 24, resulting in a model with an average F-measure of $0.78\pm 0.04$. The normalised average confusion matrix over the 300-fold cross-validation is presented on Fig. 3a. The accuracy values per class are between 0.69 and 0.91. The most confusions occur between mesophiles and psychrophiles, with psychrophiles being predicted as mesophiles in 25% of cases, and mesophiles being predicted as psychrophiles in 16%.

To identify the metabolic compounds and reactions differentiating the temperature classes, for each model estimated in the cross-validation, we selected the 50 most predictive nodes according to the Gini impurity criterion. Figure 3b shows the percentage of models that have common nodes among the 50 most predictive. There are 17 nodes common to at least 90% of models, the percentage of models sharing the same nodes decreases rapidly afterwards, with only 50% of models having node 50, and the union of 50 most useful nodes across all cross-validation models amounts to almost 160 nodes. These results display how the number of important compounds and reactions needed to distinguish the temperature classes is fairly lower than 50, with the rest of nodes being rather inconsistent. We developed and tested another approach, based on sliding masks, that leads to similar results (Supplementary Figure S1 in the supplementary data).

For each subset of 50 most predictive nodes, we also considered in which metabolic pathways these nodes appeared, by consulting the KEGG database^1^. In the table on Fig. 3d, we show these metabolic pathways, along with the average number of nodes from these 50 most important nodes, across the cross-validation. We observe that several pathways refer to amino acid metabolism and other basic metabolic pathways (pyruvate metabolism, glutathione metabolism, etc.). These findings support that temperature class differences rely on basic cell functions.

To identify which nodes and pathways are specific to each temperature class, we selected the 17 nodes common to at least 90% of models, and checked the proportion of species that have these nodes in each temperature class, which is shown on the barplot of Fig. 3c. This figure reveals that some nodes, such as *Pyridoxal 5’-phosphate synthase (glutamine hydrolysing)* (4.3.3.6_1), *ABC-type tungstate transporter* (7.3.2.6), and *Tungstate* (C20679) are more specific to species from warm habitats (hyperthermophiles and thermophiles), while others (*Glutathione disulfide* (C00127), *Gamma-L-Glutamyl-L-cysteine* (C00669), *Glutamate-cysteine ligase* (6.3.2.2), *Glutathione synthase* (6.3.2.3), ...) are clearly more specific to species from colder habitats (mesophiles and psychrophiles).

We explored the KEGG pathways of these specific nodes. The glutathione metabolism pathway (5 nodes: 6.3.2.2, C00669, 6.3.2.3, C00127, 1.8.1.7) is associated to species living in cold temperatures, and 2 nodes associated to warm temperatures belong to tungstate metabolism.

#### Growth temperature prediction

A feed-forward artificial neural network was trained in order to predict growth temperature directly (see “Methods”). In this analysis we also randomly selected 300 mesophiles and weighted the mean square objective function in order to balance the temperature distribution.

Fig. 3e displays a plot of the real growth temperature values versus the predicted ones. With an $\hbox {R}^{2} = 77.09\%$, our neural network exhibits a rather accurate prediction of the growth temperature, even with the reduced dimension that the scope entails.

### Habitat and oxygen tolerance

We applied the predictive analysis also to other environmental variables, namely, to habitat and oxygen tolerance.

We explore the habitat, a simplified variable consisting of a “Symbiont” class with 554 bacteria living in a host, an “Environment” class, with 395 free-living bacteria, and a “Mixed” class, with 235 bacteria that can live in a host or freely in the environment (see “Methods”).

We also use the oxygen tolerance information, where we have 917 prokaryotes in the “Aerobe” class, 782 in “Facultative”, and 532 in the “Anaerobe” class (see “Methods”).

**Figure 4:**
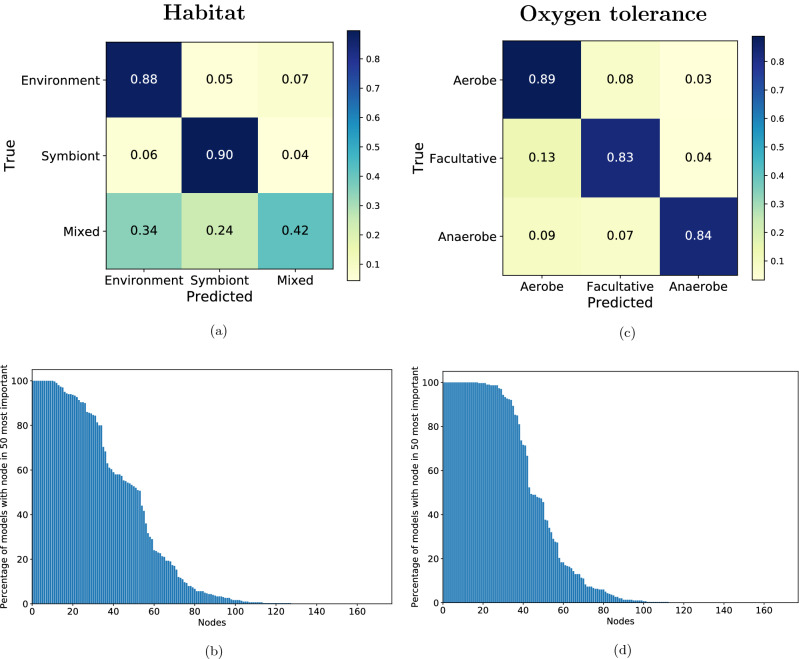
Prediction of habitat and oxygen tolerance using random forest. (**a**) Confusion matrix of a random forest habitat prediction, mean over 300-fold cross-validation. (**b**) Percentage of models (among 300 models obtained by 300-fold cross-validation) including nodes from the 50 most predictive ones, prediction of habitat using the random forest classifier. The 50 most predictive nodes were determined by the Gini impurity for each cross-validation model. (**c**) Normalised confusion matrix of a random forest oxygen tolerance prediction, mean over 300-fold cross-validation. (**d**) Percentage of models (300 models obtained by the cross-validation using the random forest) including nodes from the 50 most predictive ones, prediction of oxygen tolerance using the random forest classifier. The 50 most predictive nodes were determined by Gini impurity for each cross-validation model.

In the same way as for growth temperature classes, we built a 300-fold cross-validated weighted model for each environmental variable. The results are shown on Fig. 4.

For the habitat, the average maximal tree depth was 28, with an average F-measure of $0.80\pm 0.03$. For the oxygen tolerance, it was 31 and $0.86\pm 0.02$ respectively. Confusion matrices show reasonable results per class for oxygen tolerance (Fig. 4c), and also for habitat (Fig. 4a), with values higher than 80%. However, the exception is the “Mixed” habitat class, with accuracy of 42%, where 24% of species are misclassified as “Symbiont” and 34% as “Environment”. This problem is likely related to the definition of the “Mixed” class.

We identified the nodes of interest for the classification of the habitat and the oxygen tolerance, by choosing the 50 most predictive nodes in each fold of the cross-validation. On Fig. 4b and d, we present the percentage of models per node in the union of 50 most predictive nodes across cross-validation, for habitat and oxygen tolerance respectively. Compared to Fig. 3b, there are more nodes that are highly shared (27 nodes shared by more than 90% of cross-validation models for habitat, and 35 nodes for oxygen tolerance, whereas temperature classes had 17), meaning that the random forest needs more nodes to distinguish the different classes.

We identified the pathways to which the most important nodes belong to, and we found that the most important KEGG pathways for the habitat are aminoacid metabolism pathways (Alanine, aspartate and glutamate metabolism, Glycine, serine and threonine metabolism, Cysteine and methionine metabolism), followed by Methane metabolism and Nitrogen metabolism. For the oxygen tolerance, the pathways with the most chosen nodes are Glutathione metabolism, Carbon fixation pathways in prokaryotes, Alanine, aspartate and glutamate metabolism, and also Citrate cycle (TCA cycle).

Although we noticed that some pathways are recurrently present, since the corresponding nodes are selected frequently, some pathways, on the contrary, are specific to particular environmental variables.

## Discussion

This study introduces metabolic networks on a wide-scale of 5610 prokaryotic organisms, and discusses how it is possible to infer environmental and taxonomical information from the topology of the network.

The topological analysis of the networks presented is based on the scope of metabolic networks from the union of four chemically-defined mediums. The scope can be understood as an interpretable embedding with strict constraints, due to the rules of the scope expansion. As demonstrated by our t-SNE projections and the results of the predictive analysis, although the scope significantly reduces data dimension, the reduced data still carries sufficient information to reasonably predict the environmental and taxonomical variables: the taxonomical classification, growth temperature values and classes, habitat, and oxygen tolerance.

An important remark is that the scope is able to provide us with metabolic information helpful for the prediction of the environmental classes, and at the same time be *interpretable*. Predicting temperature classes, we identified a pathway that is prevalent in species from cold habitats —Glutathione metabolism—, and nodes prevalent in species from hot habitats are involved in tungstate metabolism.

In the case of tungstate metabolism, metabolite tungstate (C20679), and consequently ABC-type tungstate transporter (7.3.2.6), were identified as very specific to species from hot environments, especially hyperthermophiles (Fig. 3c). Tungsten is found present in higher concentrations in specific environments such as hydrothermal vents and hot-springs, compared to the open ocean. This finding is consistent with the knowledge about the environment where thermophilic and hyperthermophilic species live in^18^. Some hyperthermophilic archaea are even vitally dependent on tungsten^18^.

Glutathione (GSH) metabolism was also found to be a pathway of interest containing 5 nodes specific to cold species: glutamate-cysteine ligase (6.3.2.2), gamma-l-Glutamyl-l-cysteine (C00669), glutathione synthase (6.3.2.3), glutathione disulfide (C00127), and glutathione-disulfide reductase (1.8.1.7). These nodes constitute two glutathione biosynthesis subpathways. In the first one, Glutamate-cysteine ligase (6.3.2.2) takes aminoacids l-glutamate and l-cysteine and makes gamma-l-Glutamyl-l-cysteine (C00669). Glutathione synthase (6.3.2.3) then catalyses the reaction that transforms gamma-l-Glutamyl-l-cysteine (C00669) and glycine into glutathione.

The second glutathione biosynthesis subpathway takes glutathione disulfide (GSSG, C00127) and reduces it to glutathione, with the glutathione-disulfide reductase (1.8.1.7).

These two subpathways are not present in hyperthermophilic bacteria and archaea, with none of the species having the genes encoding the enzymes of the first one, and only 2 hyperthermophilic archaea out of 65 having the gene for the enzyme in the second subpathway (Fig. 3c).

For thermophilic species, few species have the enzymes of the first subpathway, even though the first enzyme (6.3.2.2) is slightly more prevalent in the bacterial graphs (16% of thermophilic bacteria, 5% of thermophilic archaea). The second subpathway enzyme is present in respectively 13% and 3% of thermophilic archaea and bacteria.

Multiple hyperthermophilic archaea are reported to miss glutathione (GSH and GSSG forms) in cell extracts as well as glutathione synthetase genes^19,20^, generally preferring other intracellular thiols to respond to oxidative stress, such as coenzyme A (CoA)^21,22^. Indeed, CoA has been shown to be more stable than glutathione at high temperatures^22^, and may therefore exhibit a functional role equivalent to glutathione in thermophilic and hyperthermophilic organisms^21^, explaining glutathione specificity for colder temperatures in our models. In the case of hyperthermophilic bacteria, the absence of glutathione is not as clearly shown in the state-of-the-art as for hyperthermophilic archaea, however, Hummel, Lancaster and Crane^21^ do hypothesize this, pointing to future work focused on bacteria. Our results would, therefore, bring confirmation, going in the same direction.

On another hand, glutathione is synthesized in almost all gram-negative bacteria, but it is more diversified for gram-positive bacteria^23^. It is known to be absent in some gram-positive bacteria of anaerobic or microaerophilic sources^23^, which also supports the results we found in the case of oxygen tolerance, where glutathione metabolism pathway was found to be the most important pathway to distinguish oxygen tolerance classes.

However, it is important to keep in mind that although the number of prokaryotic species studied is high, as they were taken from the complete list of species of the KEGG database, they have the same biases found in the database: a very low number of extremophiles when compared to mesophiles, a bigger number of bacteria studied than archaea, as well as some very well studied species, where multiple strains are considered, compared to less studied species. We handled this problem by subsampling mesophiles in the growth temperature analysis.

Another important remark is that the scope is a property completely based on topology, which allows its ease of use on a wide range of species, but it ignores stoichiometry and biomass dynamics. It is also strongly dependent on the input metabolites and the completeness of our networks, which greatly impacts the size of the scope. However, the results found still give sufficient information and, as we have just mentioned, are corroborated by what is found in literature.

For the sake of completeness, we tested whether the presence or absence of enzymes could also accurately predict the growth temperature using similar methods. This approach is akin to a classical differential genomics analysis without going through the network derivation and scope evaluation (albeit using only enzymes and not the whole genome, where some genes may not be annotated or code for an enzyme). In Supplementary Figure S2 of the supplementary material, we show that it displays similar prediction accuracies compared to our approach despite being of higher complexity. Moreover, our scope analysis adds comparable functional metabolic information thanks to the connectivity information, possible redundancy of pathways, and also as it is observed on the same medium. This explains why the enzymes and pathways of interest found by the random forest models for these two cases are different (see supplementary information).

In this work, we have mainly focused on a generic medium for our scope, built on different species examples from each temperature class. As it happens, it also encompasses the various oxygen tolerance classes. As a potential research avenue, it would be interesting to also test a medium based on the different habitat classes, even though it is not a trivial task, as even a single habitat class may display a large variability.

Overall, our results suggest that large scale datasets of prokaryotic species can be compared using interpretable embedding that also reduces dimensionality. Our use of the scope of metabolic networks provided key metabolites and pathways that are characteristic to environmental pressure that have somewhat been validated (or at least hinted) by wet lab experiments. This works can be extended—using other databases—to different environmental aspects and other prokaryotic communities. Our work suggests that this can be a powerful embedding tool to bridge the gap between metabolic processes and environmental impact.

## Methods

The Python code reproducing our results, as well as the networks and metadata is publicly available on GitHub.

### Data set

All information on the species was extracted from the databases in November–December 2019.

Our data set contains 5610 prokaryotic species from KEGG database^1^. This represents almost all prokaryotic species in the database as of November 2019. Among these, 301 are archaea and 5309 are bacteria. We obtained further taxonomic information from the National Center for Biotechnology Information (NCBI) Taxonomy database^24^.

A synthesis of the data set and the associated metadata can be found on Fig. 1a.

### Growth temperature

We obtained the growth temperature values for 3392 species (190 archaea and 3202 bacteria). The temperature information originates from BacDive database^25^.

Among the 3392 species with available temperature, only 693 have optimal growth temperature. For the 2699 species left, we found temperature values at which the species grow, but they are not necessarily the optimal ones. When several temperature values or an interval were given for a species, the mean of the values was taken.

Using the growth temperature, we divided our species into four temperature classes. We considered hyperthermophilic species as species whose optimal growth temperature is above $80^o\hbox {C}$, thermophilic species with the optimal temperature between $45^o\hbox {C}$ and $80^o\hbox {C}$, mesophilic species living in the temperature range between $25^o\hbox {C}$ and $45^o\hbox {C}$, and psychrophilic species with an environment inferior to $25^o\hbox {C}$. We obtained 76 hyperthermophiles (HT), 188 thermophiles (T), 2910 mesophiles (M), and 218 psychrophiles (P).

### Habitat information

We extracted the habitat information from the FusionDB database^26^, which stores functional data and metadata for 1374 bacteria. This database divides habitats into the following categories: ’Fresh water’, ’Marine’, ’Soil’, ’Other’, ’Human’, ’Host’, and ’Multi’ (a combination of the others). These categories may be further detailed with the specific habitat where the species has been found.

In order to simplify the variable so as to focus on the hypothesis that free-living and non free-living species are metabolically different, we created an ’Environment’ class, consisting of the free-living categories: ’Fresh water’, ’Marine’, ’Soil’ categories and ’Multi’ category combining the other three. The second class created was ’Symbiont’, consisting of ’Human’ and ’Host’, representing the species living in other organisms. The last class was ’Mixed’, consisting of the ’Other’ category, and the ’Multi’ categories that combine a ’Symbiont’ category and an ’Environment’ one.

We obtained 554 bacteria in the ’Symbiont’ class, 395 in the ’Environment’ class, and 235 in the ’Mixed’ class.

### Oxygen tolerance information

We acquired the species’ oxygen tolerance information from the Genomes OnLine Database (GOLD)^27^, completed with FusionDB database^26^. There are 6 different categories, from mandatory oxygen to mandatory lack of oxygen: ’Obligate aerobe’, ’Aerobe’, ’Microaerophilic’, ’Facultative’, ’Anaerobe’, ’Obligate anaerobe’. However, as classes ’Obligate aerobe’, ’Microaerophilic’, ’Obligate anaerobe’ amount to only 8% of oxygen tolerance information, we removed them. We, therefore, have data for 2103 bacteria and 128 archaea, consisting of 917 ’Aerobe’, 782 ’Facultative’, and 532 ’Anaerobe’.

### Metabolic networks

There exist a number of ways to produce metabolic networks from chemical reactions. The nodes of such a metabolic reconstruction can be metabolites (small molecules, substrates and products of the enzymes), or enzymes. The analysis in this paper is based on a representation using both metabolites and enzymes as nodes. There is a directed edge between substrates and the catalysing enzyme, and the catalysing enzyme is linked to each product.

In order to have a very extensive dataset and maintain database consistency, our networks were directly derived from the KEGG database^1^. We started with the first KEGG brite entry for each species. The brite entry lists gene names and the annotated enzyme commission (EC) codes for a given organism. From each entry we extracted all EC codes if they were found in complete form, i.e., no hyphen was present in the code. We then retrieved all Reaction Numbers (RN), found in the KEGG enzyme entries, consisting of the reactions catalysed by the enzymes. From the KEGG reaction entries, we extracted all substrates and products, and we built the directed edges between each substrate and each enzyme, and each enzyme and each product. Each reaction of a single enzyme was treated separately, with “_0”, “_1” and so on after the enzyme name to differentiate them.

Some ubiquitous metabolites present in a big number of reactions cause an important impact on a network structure. A common practice is to exclude them to make the network more relevant biologically. Indeed, very widely available and used currency metabolites such as water, ATP, ADP, and so on will greatly impact the structure in regard of network topological properties such as the path length (number of steps in a pathway)^4,28^. The average distance of the shortest paths between two metabolites is greatly shortened by the currency metabolites, since they are used in a great number of reactions, and, therefore, short circuit the actual relevant biochemical pathway when studying the network topology, making it difficult to obtain meaningful functional information. The ubiquity of these metabolites is another reason why they will not give us specific biological information, and are, therefore, commonly omitted^28^. Furthermore, removing these nodes does not separate the network into multiple subnetworks: the integrity of the network is kept for almost all reactions^28^.

There is no strict consensus on ubiquitous metabolites, but the metabolites used as electron carriers and other metabolites transferring common functional groups are usually considered as ubiquitous metabolites^29^. We used 14 ubiquitous metabolites^8^: $\hbox {H}_{2}\hbox {O}$, ATP, ADP, $\hbox {NAD}^{+}$, NADH, $\hbox {NADP}^{+}$, NADPH, $\hbox {CO}_{2}$, ammonia, sulfate, thioredoxin, phosphate, pyrophosphate ($\hbox {PP}_{\mathrm{i}}$), and $\hbox {H}^{+}$. All of these metabolites do not appear in our graphs.

The default direction of the KEGG reaction was used to orient the edges, which is the direction of the catalytic reaction. It is the direction in which the flow of biomass is expected.

### Scope of a metabolic network

The scope of a metabolic network is based on the concept of network expansion^11^. It relies on the sequential nature of chemical reactions in the metabolism. The concept is illustrated in Fig. 1b.

The expansion starts from a set of seed compounds, a medium (Fig. 1b in orange), and incrementally goes through the metabolic network if the substrates of the following enzymes are present among the products of the previous enzymes. The expansion stops if there isn’t any enzyme left with available substrates or if the whole network has been traversed.

The metabolites and enzymes of the expanded network form the scope of the network (Fig. 1b in orange and yellow). Hence, it represents the metabolic capacity of the species from the input medium.

The seed metabolites we used are metabolites from the union of four chemically-defined growth mediums used to grow a mesophilic species^14^, a psychrophilic species^15^ and a hyperthermophilic species^16^ respectively, so as to cover the diversity of our species. The thermophilic medium^17^ is already included in the union of the three other mediums. The mesophilic and hyperthermophilic species mediums were found in MediaDB^30^, a database of chemically-defined growth conditions, as it enables easy deduction of metabolites in medium.

We evaluated which nodes are in the scope for each species, and built a binary matrix providing information whether the nodes are in/out of the scope for each species. The rows of this matrix are all species of the data set, and the columns are the nodes. Species were sorted per taxonomy. The nodes were sorted according to the number of species having nodes present in scope. The nodes found in or out of the scope for all species were removed.

The algorithm implemented is based on a Breadth First Search approach, starting at each metabolite from the growth mediums. Reactions are considered successively. A reaction will be in the scope if all its substrates are, resulting in all products of the reaction also in the scope. See the algorithm in the supplementary material.

### t-SNE

We visualised the data with a t-distributed Stochastic Neighbor Embedding (t-SNE)^31^ in two dimensions. We computed the pairwise Jaccard distance between species using the nodes in scope per species. After comparing multiple values, we used a perplexity of 40, and coloured the species according to taxonomy, habitat, oxygen tolerance, and growth temperature classes (Fig. 2).

### Random forest

We built random forest classifiers^32^ to predict the growth temperature classes, habitat, and oxygen tolerance. The classes in each model were weighted according to the observation frequencies of each class. We performed a 300-fold cross-validation in order to increase the stability of our results. 10% of the species for which we have environmental information were kept as a test data set, an unbiased evaluation of the model. The other species were divided into a 66% training data set and a 34% validation data, on which the model was trained and the model parameters adjusted. The criterion for the quality of split in the decision trees was the Gini impurity. Our optimal random forest models have 1000 trees.

In the case of temperature classes, mesophiles were reduced to 300 in order to further balance the growth temperature classes. For each model learned within the 300-fold cross-validation, 300 mesophiles were randomly chosen, and the data set was then divided into training, validation and test data sets.

### Artificial neural networks

In order to predict not only the growth temperature class, but also the growth temperature values, we built a feed-forward neural network.

We again selected 300 random mesophiles to reduce the temperature classes unbalance. 66% of species were used as the training data, and 34% for test. We normalised the growth temperature values (T) as follows: $T_{norm} = \frac{T - T_{min}}{T_{max}-T_{min}}$.

Our network has three layers: an input layer, with as many input neurons as the number of compounds, a hidden layer of size 1000, and an output layer with a single neuron, which returns the predicted growth temperature. The hidden layer takes as input a linear function of the weights and input values, coupled with a dropout of probability 0.2 to prevent overfitting^33^. The output neuron takes the output of the hidden layer, multiplies it by the weights, and applies a sigmoid function.

The weights are found by optimizing the mean squared loss with the Adam optimizer^34^. We developed our own mean squared loss function to account for the unbalanced temperature distribution. We applied a learning rate of $1\mathrm {e}{-4}$ for 250 epochs (cycles through the training set). All hyper-parameters such as the number of layers, neurons, and epochs are fixed using the cross-validation. We used the pytorch (https://pytorch.org/) framework to declare and train the network.

## Supplementary Information

Supplementary Information.

## Data Availability

The open source code (Python) reproducing our results is publicly available at: https://github.com/AWebZen/FunctionalPrediction5000species. The repository also contains the generated networks (in form of adjacency matrices), and metadata.
