# Supplementary material for: Functional prediction of environmental variables using metabolic networks
Source: Sci Rep. 2021 Jun 9;11:12192. doi: 10.1038/s41598-021-91486-8 (PMC8190111; doi:10.1038/s41598-021-91486-8)
Supplement: Supplementary file 1 — Supplementary Information. [file 41598_2021_91486_MOESM1_ESM.pdf]

Supplementary information:

Functional prediction of environmental variables using metabolic networks

Adèle Weber Zendrera[1,2], Nataliya Sokolovska[1,2], and Hédi A. Soula[1,2]

[1]Sorbonne University, Paris, 75005, France
[2]INSERM UMRS1269 NutriOmics, Paris, 75013, France
*adele.weber@etu.sorbonne-universite.fr

Scope of a metabolic network algorithm

You will find below the algorithm for the scope that we implemented.

Algorithm 1: Scope algorithm

Input: Input growth medium $G \subset M$
Metabolic network $N = (R, M, E)$ (prior knowledge)
//R and M are sets of nodes representing Reactions and Metabolites
//E denotes Edges linking substrates to the reactions and reactions to products

Output: scope $\Sigma(G, N) \subset \{M, R\}$

Initialize: $\Sigma(G, N) = G$ *// The scope contains the growth medium*

```
# Pseudocode
 for g in G:
     for reaction in Breadth_First_Search(g):
         substrates = graph.predecessors(reaction)
         if substrates ⊂ Σ(G,N):
             Σ(G,N) = Σ(G,N) ∪ reaction
             products = graph.successors(reaction)
             Σ(G,N) = Σ(G,N) ∪ products
```

Alternative identification of important nodes: decrease of accuracy

In order to identify nodes of interest to distinguish classes in our random forest models, we compared the results of the Gini impurity with another technique based on a decrease of accuracy. To test this, the same kind of random forest model as in Figure 3 was built for the optimal growth temperature classes, but with a 102-fold cross-validation instead of 300 folds, due to its slower speed.

In this technique, we take a window of a definite size —50 nodes in our case— that we slide across our nodes in the matrix of species per nodes in scope. We will be working on our testing data set. The values of the matrix in this window of nodes will be inverted: if a species has one of the specific nodes in its scope, the node will be put as out of scope, and vice versa. The accuracy of the model prediction will be re-evaluated on the new matrix. The position of the window with the largest decrease in accuracy when compared to the accuracy without any window modification will contain the most important nodes.

The average global accuracy of the model without alterations is of $77.58\% \pm 5.07\%$. Supplementary Figure SS1a shows the number of cross-validation folds with the window with most impact on accuracy at a certain position (if multiple positions had the same impact, all are represented). These results show that nodes in the windows that start between 150 and 180 are most commonly the windows with the largest decrease of accuracy.

In Supplementary Figure SS1b, each point in black shows the accuracy of the window with greatest impact on accuracy per cross-validation fold, at the first node position of the window. In green, we see the first 100 nodes that are commonly considered across cross-validation folds as very important by the Gini criterion. Therefore, the abscissas of the black points actually represent nodes in positions from x to $x + 49$, whereas the points in green represent only the node that is found at position x.

As the windows of nodes starting at the black points cover the green Gini nodes, we considered that the nodes of importance found by both techniques are substantially similar, thus the Gini feature importance tool was chosen because of its greater speed.

Enzyme Commission random forest prediction of growth temperature classes

We also looked at the results of a random forest prediction of growth temperature classes by taking the list of enzymes (unique Enzyme Commission EC codes) present or absent in the metabolic network of each species, instead of the metabolic scope. The total number of enzymes considered is 2848.

Our approach of taking EC codes comes close to a differential genomics analysis, except that it only denotes genes annotated with an EC code, and also we don't take into account the fact that some genes may have several ECs associated.

The same protocol as for the scope was used to build the random forest classifier, with only the input matrix being different (see Methods).

Supplementary Figure SS2 shows the results of the growth temperature prediction. The average maximal tree depth is 24. The average F-measure is $81.35\% \pm 4.27\%$ compared to $78.27\% \pm 4.39\%$ for the scope based. Accuracies per class are very high (supplementary Figure SS2a), slightly higher than the ones found with the scope-based prediction (Figure 3a), especially for psychrophiles, which are predicted correctly for 75% of species, and 69% with the scope-based prediction.

Supplementary Figure SS2b shows the percentage of folds in cross-validation where a certain node has been identified as among 50 most important nodes in prediction. Few nodes are actually consistently common across folds, with only 16 nodes present in mode than 90%, which is equivalent to what is needed in the scope-based random forest prediction.

However, the KEGG pathways to which the enzymes belong to are very different than the ones from the scope-based prediction: Cysteine and methionine metabolism, Methane metabolism, and Lipopolysaccharide biosynthesis.

In this analysis we only consider genes annotated as enzyme but results are very similar in accuracy to what we obtain using the scope despite the reduction of dimension. Using the scope, we obtain reaction information that contextualises enzymes in a metabolic pathway associated with the surrounding metabolites, accounting for pathway redundancy, and in response to the given medium. Although accuracies are similar, pathway information is different and contextual.

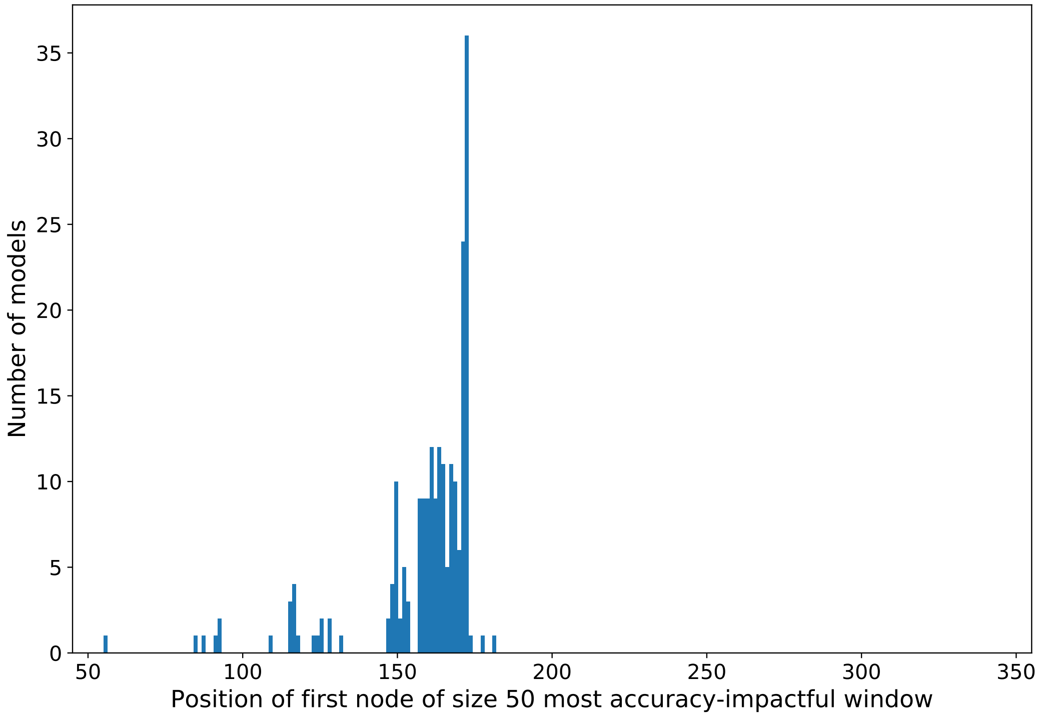

(a) Histogram of the number of models per position (first index) of the window of size 50 with the most impact on accuracy.

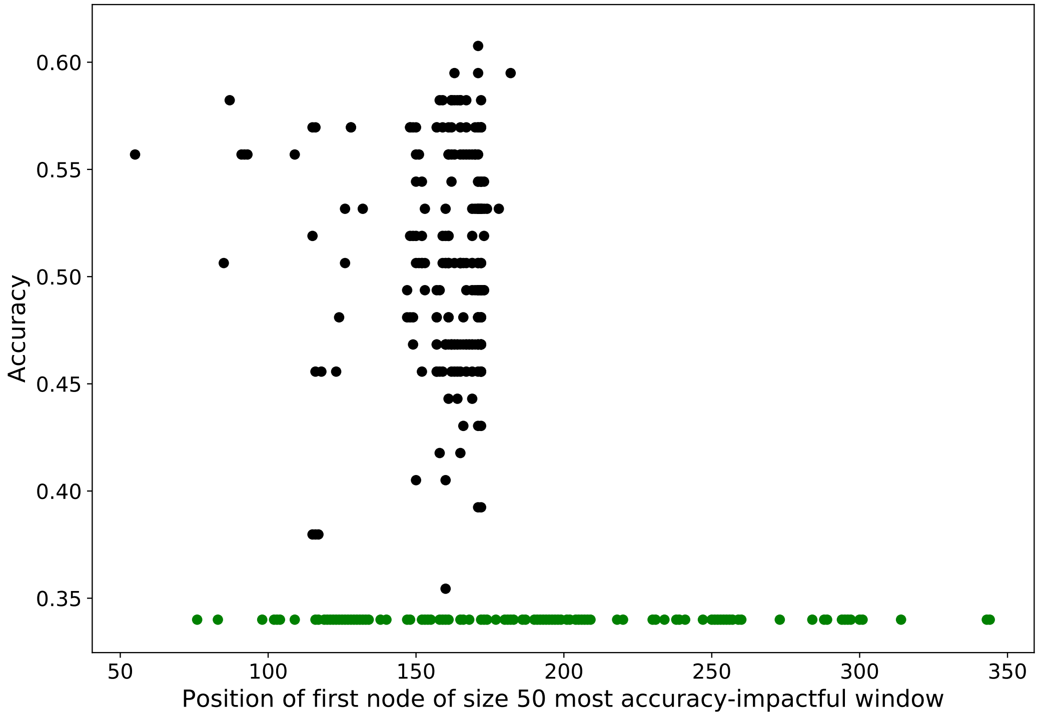

(b) Accuracy of prediction for inversion window with most impact per cross-validation fold. X-axis is the position (first index) of the inversion window of size 50 with the most impact on accuracy. In green, the position of the first 100 most common nodes amongst the 50 most important Gini nodes across cross-validation, at an arbitrary ordinate.

Figure S1. Decrease of accuracy vs Gini impurity analysis

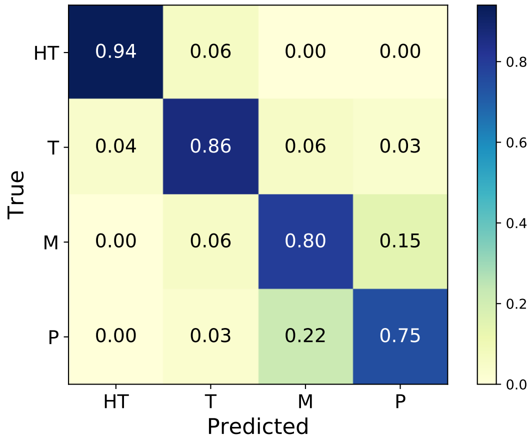

(a)

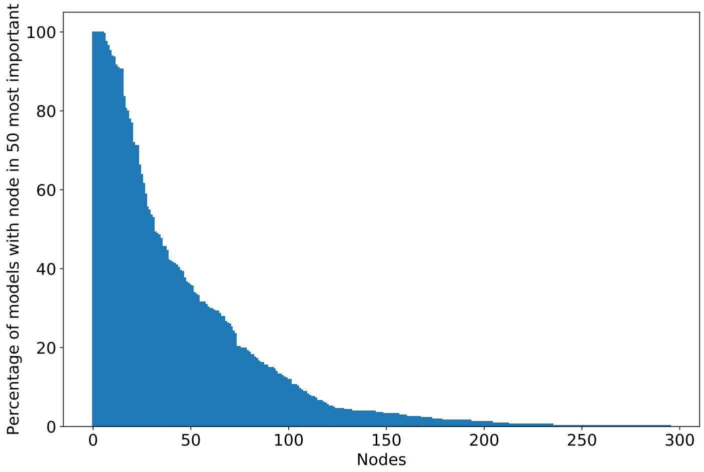

(b)

Figure S2. Growth temperature classes random forest classification from Enzyme Comission presence (a) Normalised confusion matrix of a growth temperature class random forest prediction, mean over 300-fold cross-validation. (b) Percentage of models with node in 50 most important nodes, among 300-fold cross-validation of growth temperature class random forest classifier. 50 most important nodes determined by Gini impurity for each cross-validation model.
